# TUNAR lncRNA Encodes a Microprotein that Regulates Neural Differentiation and Neurite Formation by Modulating Calcium Dynamics

**DOI:** 10.3389/fcell.2021.747667

**Published:** 2021-12-31

**Authors:** Elena Senís, Miriam Esgleas, Sonia Najas, Verónica Jiménez-Sábado, Camilla Bertani, Marta Giménez-Alejandre, Alba Escriche, Jorge Ruiz-Orera, Marta Hergueta-Redondo, Mireia Jiménez, Albert Giralt, Paolo Nuciforo, M. Mar Albà, Héctor Peinado, Daniel del Toro, Leif Hove-Madsen, Magdalena Götz, María Abad

**Affiliations:** ^1^ Cellular Plasticity and Cancer Group, Vall d'Hebron Institute of Oncology (VHIO), Barcelona, Spain; ^2^ Physiological Genomics, Biomedical Center (BMC), Helmholtz Center Munich, Institute of Stem Cell Research, Großhaderner Str, SyNergy Excellence Cluster, Ludwig-Maximilians-Universitaet (LMU), Munich, Germany; ^3^ Instituto de Investigación Biomédica Barcelona (IIBB-CSIC), Instituto de Investigación Biomédica Sant Pau (IIB-Sant Pau) and CIBERCV, Barcelona, Spain; ^4^ Cardiovascular and Metabolic Sciences, Max Delbrück Center for Molecular Medicine in the Helmholtz Association (MDC), Berlin, Germany; ^5^ Microenvironment and Metastasis Laboratory, Molecular Oncology Programme, Spanish National Cancer Research Center (CNIO), Madrid, Spain; ^6^ Department of Biological Sciences, Institute of Neurosciences, IDIBAPS, CIBERNED, University of Barcelona, Barcelona, Spain; ^7^ Molecular Oncology Group, Vall d'Hebron Institute of Oncology (VHIO), Barcelona, Spain; ^8^ Evolutionary Genomics Group, Research Programme on Biomedical Informatics, Hospital del Mar Medical Research Institute (IMIM) and Universitat Pompeu Fabra (UPF), Barcelona, Spain; ^9^ Catalan Institution for Research and Advanced Studies (ICREA), Barcelona, Spain

**Keywords:** microproteins, micropeptides, sORF encoded peptides, TUNAR, neural differentiation, neurite formation, calcium, long non-coding RNAs

## Abstract

Long noncoding RNAs (lncRNAs) are regulatory molecules which have been traditionally considered as “non-coding”. Strikingly, recent evidence has demonstrated that many non-coding regions, including lncRNAs, do in fact contain small-open reading frames that code for small proteins that have been called microproteins. Only a few of them have been characterized so far, but they display key functions in a wide variety of cellular processes. Here, we show that TUNAR lncRNA encodes an evolutionarily conserved microprotein expressed in the nervous system that we have named pTUNAR. pTUNAR deficiency in mouse embryonic stem cells improves their differentiation potential towards neural lineage both *in vitro* and *in vivo*. Conversely, pTUNAR overexpression impairs neuronal differentiation by reduced neurite formation in different model systems. At the subcellular level, pTUNAR is a transmembrane protein that localizes in the endoplasmic reticulum and interacts with the calcium transporter SERCA2. pTUNAR overexpression reduces cytoplasmatic calcium, consistent with a possible role of pTUNAR as an activator of SERCA2. Altogether, our results suggest that our newly discovered microprotein has an important role in neural differentiation and neurite formation through the regulation of intracellular calcium. From a more general point of view, our results provide a proof of concept of the role of lncRNAs-encoded microproteins in neural differentiation.

## Introduction

Long noncoding RNAs (lncRNAs) are RNA molecules of more than 200 nucleotides with diverse regulatory roles, which have been long assumed to not code for proteins. However, recent findings indicate that some lncRNAs and other assumed non-coding regions, such as microRNA precursors, introns and untranslated regions (UTRs) of coding transcripts actually contain small-open reading frames (sORFs) that are translated into bioactive small proteins (Makarewich and Olson 2017; Orr et al., 2020; Peeters and Menschaert 2020). These sORFs have been long ignored mainly due to an arbitrary cut-off of 300 nucleotides set by ORF prediction algorithms. Therefore, proteins smaller than 100 amino acids have been systematically overlooked. The advances in ribosome profiling and proteomics have allowed the discovery and characterization of a wide variety of small proteins of less than 100 amino acids in length that have been called microproteins, micropeptides or SEPs (sORF-encoded peptides) (Merino-Valverde et al., 2020). Microproteins have been shown to play key functions in a plethora of cellular processes, spanning calcium dynamics (Anderson et al., 2016), mRNA turnover (D'Lima et al., 2017), DNA repair (Slavoff et al., 2014), mitochondrial function (Stein et al., 2018) and lipid metabolism (Polycarpou-Schwarz et al., 2018), among others. Given the large number of predicted sORF-peptides (in the order of tens of thousands), this might just be “the tip of the iceberg” and there might be hundreds of undescribed microproteins with important roles in diverse cellular processes. Importantly, the relevance of the microproteome regulating neuronal processes has not yet been revealed.

TUNAR is a long non-coding RNA initially described in zebrafish with the name *Megamind* (Ulitsky et al., 2011). It is expressed at very early stages of embryonic development (from the preimplantation embryo until the blastocyst stage) (Bouckenheimer et al., 2018) and in adults it is mainly expressed in the brain, particularly in neurons and newly formed oligodendrocytes (Dong et al., 2015). Of note, TUNAR has been shown to maintain pluripotency and promote neural lineage commitment (Lin et al., 2014). Specifically, TUNAR lncRNA forms a complex with three RNA binding proteins (RBP) and this TUNAR-RBP complex binds to the promoters of the pluripotency master genes Nanog and Sox2, as well as to the promoter of the neural differentiation factor Fgf4 (Lin et al., 2014). Consistent with its pro-differentiation role, TUNAR is downregulated in glioblastoma multiforme (Reon et al., 2016).

Here, we describe that TUNAR lncRNA encodes a 48 amino-acid microprotein expressed in the nervous system that we have named pTUNAR. Parallel to our work, pTUNAR, under the name of beta cell- and neural cell-regulin (BNLN), has been recently described to be expressed in pancreatic ß cells contributing to their function (Li et al., 2021). In our work, we show that pTUNAR deficiency promotes neural differentiation of mouse embryonic stem cells (mESCs) *in vitro* and *in vivo*. Moreover, pTUNAR overexpression impairs neurite outgrowth in different models of neuritogenesis. At the subcellular level, pTUNAR localizes in the endoplasmic reticulum (ER) membrane, interacts with SERCA2 and modulates calcium exchange between the ER and the cytosol. Our findings underscore the importance of the microproteome as a source of previously undescribed regulators of neural differentiation.

## Materials and Methods

### General Cell Culture

HEK293T and NIH3T3 cells were cultured in DMEM with GlutaMAX supplemented with 10% of fetal bovine serum (FBS) and 1% of Penicillin-Streptomycin (P/S) (Gibco). Neuro-2a (N2A) (ACC 148, German Collection of Microorganisms and Cell Cultures) cells media was further supplemented with non-essential aminoacids (NEAA). Mouse embryonic stem cells (mESCs) v6.4 were cultured in DMEM GlutaMax supplemented with 1% Sodium Pyruvate (Invitrogen), 15% FBS, 50 mM ß-mercaptoethanol, 1% NEAA (Invitrogen), 1% P/S and 1000 U/ml LIF (ESGRO, Chemicon).

### *In vitro* Differentiation Experiments

#### Embryoid Bodies Differentiation

mESCs were cultured in hanging drops (1,000 cells/20 µL) prepared with mESCs media without LIF for 3–4 days. Then, the already formed embryoid bodies were transferred to suspension culture (in 10 cm^2^ Petri dishes) and were cultured in mESC media without LIF for 10 more days.

#### Neural Differentiation of Mouse Embryonic Stem Cells

Mouse embryonic stem cells were differentiated to neurons using the protocol previously described (Mao and Zhao, 2020). Briefly, 1.5 × 10^6^ cells were seeded in suspension (10 cm^2^ Petri dishes) with Basal Differentiation Media I for 2 days. Then, the embryoid bodies (EBs) formed were transferred to gelatin-coated 6 well plates (approximately 50 EBs/well) and cultured in Basal Differentiation Media I supplemented with 1 µM retinoic acid (Sigma) for 6 days. At that point, cells were counted and 5 × 10^5^ cells were seeded in gelatin-coated 6 well plates. The next day, medium was changed to N2B27 medium II and cells were cultured for 10 more days. N2B27 medium II was replaced every 2 days.

#### N2A Differentiation

N2A cells were seeded at 6 × 10^5^ cells/well in 12 well plates (Ma et al., 2015) with poly-lysine coated coverslips. The next day the media was changed to DMEM with GlutaMAX supplemented with 1% FBS, 1% of P/S and 1% NEAA. The media was changed daily and the cells processed after 72 h for immunfluorescence.

#### E13 Cortex Primary Cultures

E13 mice cortices were dissected removing the ganglionic eminence, the olfactory bulb, the hippocampal anlage, and the meninges, and cells were mechanically dissociated with a fire polish Pasteur pipette. Cells were then seeded onto poly-d-lysine-coated glass coverslips in DMEM-GlutaMAX with 10% FBS (Life Technologies). Plasmid transfection was done with Lipofectamine 2000 (Life technologies) according to manufacturer’s instruction. To be able to analyze the number of cells in S-phase, Bromodeoxyuridine-5-bromo-2′-deoxyuridine (BrdU) was added to the medium for 30 min prior to fix the cells. For differentiation analysis, cells were collected 7 days after transfection, washed in phosphate-buffered saline (PBS) and fixed in 4% paraformaldehyde (PFA) in PBS and processed for immunostaining.

### Cloning Procedures

pTUNAR ORF was synthesized (IDT technologies) fused with a flexible linker (GGGGSGGGGSGGGGS) and an HA tag epitope at the C-terminal part of the microprotein and flanked by EcoRI enzyme restrictions sites at both ends. After enzymatic digestion, constructs were ligated into the pENTR1A vector or the pMSCV vector. For the lentiviral vectors, the pTUNAR-HA tag construct was obtained by recombining donor vectors with the lentiviral inducible system pINDUCER20 (Invitrogen) or the pLV-CAG system using the Gateway Cloning Technology, following manufacturer’s instructions.

### Retro- and Lentiviral Vectors Production

#### Procedure

HEK293T cells were transfected with the lenti- or retroviral plasmids and the packaging plasmids indicated below using Fugene HD (Promega) following manufacturer’s instructions. Viral supernatants were collected twice a day on two consecutive days, filtered through a 0.45 µm syringe filter, supplemented with of 8 μg/ml of polybrene and used to infect NIH3T3 (inducible lentiviral vectors), N2A cells (constitutive lentiviral vectors) or mESCs (retroviral vectors). Successfully infected cells were established by geneticin selection (inducible lentiviral vectors), puromycin selection (retroviral vectors) or GFP expression (constitutive lentiviral vectors).

#### Plasmids Used

##### Inducible Lentiviral Vectors

pInducer-Empty (control) or pInducer-pTUNAR and packaging plasmids pLP-1, pLP-2 and pLP-VSVG.

##### Constitutive Lentiviral Vectors

pLV-CAG-GFP (control) or pLV-CAG-pTUNAR-IRES-GFP and packaging plasmids pLP-1, pLP-2 and pLP-VSVG.

##### Retroviral Vectors

pMSCV-Empty (control) or pMSCV-pTUNAR and packaging plasmid pCL-Eco.

### Generation of pTUNAR-KO Mouse Embryonic Stem Cells

mESCs cells were co-transfected with pSpCas9(BB)-2A-Puro plasmid (Plasmid #62988, Addgene) containing the sgRNAs mTUNAR1 targeting the start codon of pTUNAR (ACC​AAG​ATG​GTA​ATC​ACG​AG) and a single stranded DNA as homologous recombination template (CTTCACTACAGGTTAGCCTGGAGAGGAAGATAAAGACATTTGCAACCAAGTGAGTAATCACGAGTGGAAACGATGAAGACCGGGGAGGCCAAGAGAAAGAGAG) with Lipofectamine Stem Transfection Reagent (ThermoFisher) following manufacturer’s instructions. 24 hours after transfection, puromycin was added to select for transfected cells. pTUNAR KO single colonies were picked, expanded and its DNA was extracted and purified. The locus was PCR-amplified (Primer_mTUNAR1_F, CAA​AAC​CCC​AGC​CAG​TAC​AC; Primer_mTUNAR1_R, ATG​CAA​TGC​CTG​TCA​ACG​AA) and sent for sequencing. A clone with a homozygous substitution of pTUNAR start codon was used in this study.

### Extracellular Vesicles Purification

NIH3T3 cells were cultured in medium supplemented with 10% EV-reduced FBS (FBS, Hyclone). FBS was reduced of bovine EVs by ultracentrifugation at 100,000 × g for 70 min. Supernatant fractions collected from 72 h cell cultures were pelleted by centrifugation at 500 *g* for 10 min. The supernatant was centrifuged at 12,000 × g for 20 min to purify large extracellular vesicles (Witwer and Théry, 2019). Small extracellular vesicles (sEVs) (Witwer and Théry, 2019) were then harvested by centrifugation at 100,000 × g for 70 min. The supernatant (conditioned medium) was collected and used as control. The sEV pellet was resuspended in 20 ml of PBS and collected by ultracentrifugation at 100,000 × g for 70 min. All spins were performed at 10°C using a BECKMAN Optima X100 centrifuge with BECKMAN TYPE 70Ti rotor. EVs were resuspended in PBS and the protein content was measured by bicinchoninic acid assay (BCA) (Pierce).

### Animal Experiments

All experimental procedures involving animals in this study were reviewed and approved by the Vall d'Hebron Ethics Committee and the Commission of Animal Experimentation of Generalitat de Catalunya (Spain) and the Government of Upper Bavaria (ROB/Regierung von Oberbayern) (Germany).

#### Teratoma Formation

2 × 10^6^ of v6.4 WT, pTUNAR KO or pTUNAR OE mESCs were subcutaneously injected into the flanks of 8-week-old immunocompromised NMRI mice. Tumor growth was monitored every 2 days using the formula height × width × width × (3.1416/6) and animals were sacrificed when tumors reached 1.5 cm^3^.

#### *In uterus* Injection

E13 timed pregnant mice were anaesthetized by intraperitoneal (i.p.) injection of Fentanyl (0.05 mg/kg), Medetomidine (0.5 mg/kg) and Midazolam (5 mg/kg). LV-Piscis-IRES-eGFP and RV-RFP vectors were mixed with 0.1% Fast Green (Sigma) and 1 μL of the mix was injected in the embryo ventricle. Anesthesia was terminated by subcutaneous injection of Buprenorphine (0.1 mg/kg), Flumazenil (0.5 mg/kg) and Atipamezole (2.5 mg/kg), and the mice were allowed to recover on a heating pad and were closely monitored for proper recovery. The brains were collected 10 days after birth and used for further analysis.

### Ribosome Profiling Analysis

We retrieved a public ribosome profiling dataset from human and mouse brain (ArrayExpress accession number E-MTAB-7247) (Wang et al., 2020) and we adapted a computational approach to identify translated sORFs (Ruiz-Orera et al., 2018). In brief, read adapters were trimmed and reads mapping to annotated ribosomal and transfer RNAs were filtered out. Resulting reads were mapped to the assembled mouse genome (mm10) and human genome (hg38). Next, mapped reads from experimental replicates were merged and we used the ribORF algorithm (Ji et al., 2015) to predict translated sORFs with significant read uniformity and frame periodicity (score ≥0.7), as this feature is indicative of active ORF translation. To calculate the translational efficiency (TE) we obtained the FPKMs of the Ribo-seq and RNA-seq data sets and made the ratio of both FPKMs. Only genes with an FPKM ≥1 in RNA-seq were selected for this calculation.

### Analysis of Gene Expression by qRT-PCR

Total RNA was extracted with Trizol (Invitrogen) following manufacturer’s protocol. Genomic DNA was cleaned up and retrotranscription performed using the iScript gDNA Clear cDNA Synthesis Kit (BioRad). Gene expression was analyzed by qRT-PCR using PowerUp SYBR Green Master Mix (Thermo Fisher Scientific) in the 7900HT Fast Real-Time PCR System (Applied Biosystems). Cycle threshold (Ct) values were normalized to GAPDH. Gene-specific primers are listed in Supplementary Table S1. Of note, TUNAR lncRNA expression was determined using primers mTUNAR_F and mTUNAR_R, which do not detect exogenous pTUNAR. Exogenous pTUNAR expression was determined using a forward primer binding the endogenous locus (mTUNAR1_qPCR_F) and a reverse primer binding the HA tag sequence (HAtag_qPCR_R) (Supplementary Table S1).

### Western Blotting

Cells and tissues were homogenized in 2% SDS lysis buffer (50 mM Tris-HCl, pH 8.0, 1 mM EDTA, 2% SDS) supplemented with protease (Roche) and phophatase (Sigma-Aldrich) inhibitors cocktails. Protein concentration was determined using the Pierce™ BCA Protein Assay Kit (Thermo Fisher). Lysates were loaded in 12% bis-tris acrylamide gels and transferred to nitrocellulose membranes. Primary antibodies were incubated overnight at 4°C. Secondary HRP-conjugated antibodies were incubated the following day for 1 h at room temperature, and ECL Prime Western Blotting Detection Reagent (Fisher Scientific) or SuperSignal™ West Dura Extended Duration Substrate were used as a chemiluminescent reagent for protein detection. Antibodies and dilutions are listed in Supplementary Table S2.

### Immunoprecipitation

NIH3T3 cells were lysed in a buffer containing 50 mM Tris-Hcl pH 7.5–8, 150 mM NaCl, 1%Triton X-100 and protease and phosphatase inhibitors and homogenized for 30 min in a rotor wheel. 3 mg of lysates were immunoprecipitated with 5 μg of monoclonal HA-tag antibody (Sigma) overnight at 4°C. Immunocomplexes were collected using PureProteome™ Protein A Magnetic Beads (MERCK) and eluted by boiling for 5 min in SDS-loading buffer. Western blotting, as described above, was used to visualize pTUNAR (HA-tag antibody) and SERCA2 (SERCA2 ATPase antibody). Antibodies and dilutions are listed in Supplementary Table S2.

### Immunofluorescence

Primary antibodies and dilutions are listed in Supplementary Table S2. All secondary antibodies were purchased from Life Technologies.

#### NIH3T3 and N2A Cells

NIH3T3 and N2A cells were fixed in 4% paraformaldehyde for 15 min and permeabilized with 0.5% Triton X-100 for 7 min at room temperature. Cells were blocked in 3% Bovine Serum Albumin (BSA) and 10% goat serum in PBS for 1 hour and incubated overnight at 4°C with the primary antibody diluted in 3% BSA in PBS. Next day, secondary antibodies were incubated for 1 hour at room temperature in the dark. Cells were mounted in Prolong Mounting Medium with DAPI (Invitrogen). Images were taken in a Nikon C2 Plus Confocal Microscope.

#### Adult and Embryonic Brain

For adult brain immunostaining, paraffin blocks were sliced into 3 µm sections and incubated at 56°C overnight. The next day, tissue sections were deparaffinized with xylene (Fisher Scientific, Waltham, MA, United States) and rehydrated with decreasing concentrations of ethanol in water. Antigen retrieval was performed with boiling sodium citrate buffer at pH 6 for 7 min and sections were permeabilized with 1% Tween in PBS for 15 min. Embryonic brain cryosections were permeabilized with 0.5% Triton X-100 for 30 min. Adult and embryonic brain sections were blocked with PBS, 0.2% BSA, 5% goat serum, 0.2% glycine and 0.2% lysine for 2 hours at room temperature. Primary antibodies were diluted in PBS, 0.3% Triton X-100 and 2% goat serum and secondary antibodies in PBS, 0.3% Triton X-100 and 3% goat serum. Sections were mounted in Prolong Mounting Medium with DAPI (Invitrogen). Immunofluorescence images were taken with a C2 confocal microscope (Nikon) at ×20 magnification and applying optimal digital zoom calculated with the Nyquist-Shannon sampling theorem. Overviews of the adult and mouse brain were taken with a Leica Thunder microscope.

#### P10 Brain Sections and E13 Cortex Primary Cultures

For immunostainings in p10 brain sections and E13 primary cultures, tissue sections (20 μm) or cells plated on poly-d-lysine-coated glass coverslips were blocked with 2% BSA, 0.5% Triton-X (in PBS) for 1 h prior to staining. Primary antibodies were applied in blocking solution overnight at 4°C. Fluorescent secondary antibodies were applied in blocking solution for 1 h at room temperature. DAPI (4’,6-diamidin-2-phenylindol, Sigma) was used to visualize nuclei. Sections were mounted in Aqua Polymount (Polysciences). Images were taken using an Olympus FV1000 confocal laser-scanning microscope using 20×/0.85 N.A and 63×/1.35 NA. water immersion objective or epifluorescence microscope (Zeiss, Axio ImagerM2) equipped with a 20×/0.8 N.A and 63×/1.25 NA. oil immersion objectives.

#### Scholl Analysis

Sholl analysis was performed on cortical neurons from E13 embryos transfected with a pTUNAR-IRES-GFP plasmid or a GFP control plasmid and differentiated for 7 days. Briefly, neurons were traced using the Simple Neurite tracer plugin (v3.1.6) for Fiji (v1.52p) and their skeletonized path was rendered as binary image. Intersections with the Scholl radii were counted every 20 µm and plotted against the distance from the center of mass of the soma using the Sholl analysis plugin (v4.0.0.) for Fiji (v1.52p). Scholl radii analysis ended at 500 µm.

### Immunohistochemistry

Immunohistochemistry was performed on paraffin-embedded mouse tissues. In brief, paraffin blocks were sliced into 3 µm sections, deparaffinized with xylene (Fisher Scientific, Waltham, MA, United States) and rehydrated with decreasing concentrations of ethanol in water. Sections underwent antigenic exposure process into the Discovery Ultra (Ventana) system with CC1 buffer for 64 min at 95°C. Anti-pTUNAR antibody was incubated for 1 h at RT (Supplementary Table S2). Next, slides were incubated with the secondary antibody Discovery UltraMap anti-Rabbit HRP (Ventana). Hematoxilin and eosin staining was performed on 5 μm paraffin sections in a Robust carousel tissue stainer (Slee Medical) according to common method.

### Calcium Measurements

To measure cytoplasmic calcium, cells were incubated with 5 µM Rhod-2 (Invitrogen) for 40 min at RT. Then, cells were washed twice with a physiological buffer containing 132 mM NaCl, 0.33 mM NaH_2_PO_4_, 4 mM KCl, 4 mM NaHCO_3_, 2 mM CaCl_2_, 1.6 mM MgCl_2_, 10 mM HEPES, 5 mM glucose and 5 mM pyruvic acid. pH was adjusted to 7.4 with NaOH and experiments were done at 37°C. To release calcium from the ER, cells were superfused with 20 mM caffeine during the indicated times. Images (512 × 512 pixels) were recorded with a resonance scanning confocal microscope Leica SP5 AOBS and a HCX PL APO CS 20.0 × 0.70 IMM objective at a frame rate of 15 images/s. Images were analyzed with Leica LAS AF Software and calcium signals from individual cells in cultures were analyzed as described previously (Molina et al., 2016). The decaying phase of the calcium transient was fitted with an exponential function indicated below during caffeine exposure and after caffeine removal.

$$f\left(x\right)=y_{0}+Ae^{-t/\mathrm{tau}}$$

### *In silico* Tools

TUNAR coding potential was assessed using PhyloCSF (Lin et al., 2011), a comparative genomics method that analyzes a multispecies nucleotide sequence alignment based on a formal statistical comparison of phylogenetic codon models.

Protter (Omasits et al., 2014) was used to visualize pTUNAR predicted topology. GPS-Sumo 2.0 (Ren et al., 2009; Zhao et al., 2014) was used to predict sumoylation sites and SUMO-binding motifs. UbPred (Radivojac et al., 2010) was used to predict residues prone to be ubiquitinated. The glycomics tools from Expasy (https://www.expasy.org/) were used to predict sites of glycosylation.

Image analysis was performed using Fiji (Schindelin et al., 2012). To quantify the neurites observed in different fields and the proportion of neurites/neuron the plugin NeuronJ was used as previously described (Pemberton et al., 2018). The colocalization analysis tools from Fiji were used to calculate the co-localization of pTUNAR with SERCA2 and their co-localization coefficient. Graphs and statistical analysis were performed using GraphPad Prism 6.

### Statistical Analysis

Data is expressed as the mean ± standard deviation (SD) or the mean ± standard error of the mean (SEM) for *in vivo* experiments. Differences between groups were analyzed using one-way ANOVA, two-way ANOVA or unpaired Student’s t-test, as specified. *p*-value < 0.05 was considered statistically significant. All statistical tests were two-sided and performed using GraphPad Prism (GraphPad Software Inc., San Diego, CA, United States).

## Results

### The lncRNA TUNAR Encodes a 48-Amino Acid Microprotein Expressed in the Nervous System

**FIGURE 1:**
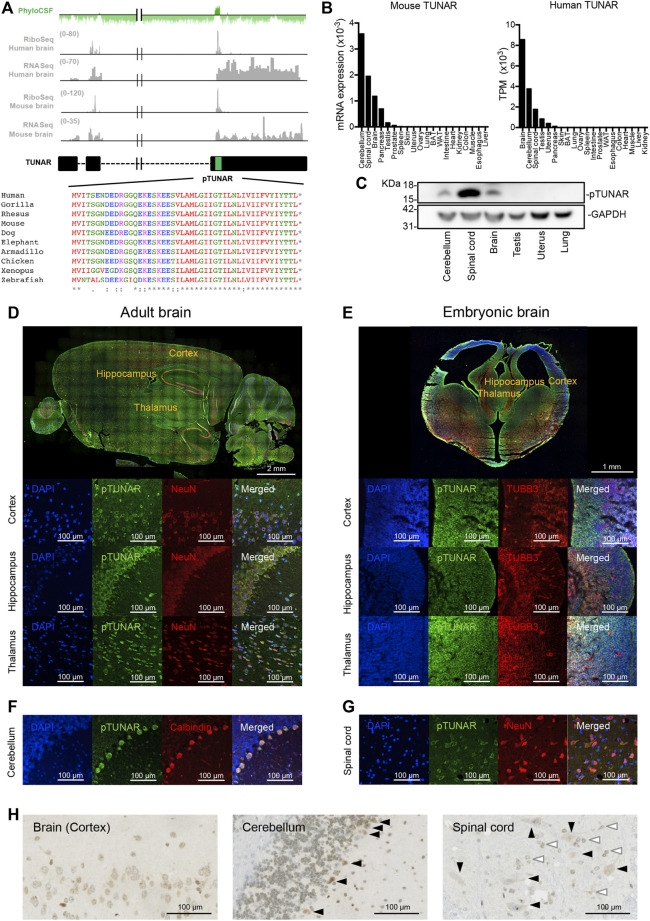
pTUNAR identification and analysis of its expression. **(A)** In green, PhyloCSF score across TUNAR gene. In grey, Ribo-seq and RNA-seq analysis of TUNAR transcript in human and mouse brain. In black, diagram of TUNAR locus. The sORF encoding pTUNAR is indicated with a green box. Below, pTUNAR amino acid conservation across vertebrates. **(B)** Bar plot showing the expression of TUNAR in different human organs (GTEx data) and in mouse organs, measured by qRT-PCR and normalized to GAPDH (data from 3 female and 3 male mice, 9 weeks old). **(C)** Western blot of endogenous pTUNAR in the indicated mouse adult organs (9 weeks old). **(D)** Immunofluorescence image showing the expression of pTUNAR and NeuN in the adult mouse brain (12 weeks old). **(E)** Immunofluorescence image showing the expression of pTUNAR and TUBB3 in E15.5 mouse cortex, hippocampus and thalamus. **(F)** Immunofluorescence images showing the expression of pTUNAR and Calbindin (Purkinje cells) in the adult mouse cerebellum (12 weeks old). **(G)** Immunofluorescence image showing the expression of pTUNAR and NeuN in the adult mouse spinal cord (12 weeks old). **(H)** Immunohistochemistry of pTUNAR in the adult mouse brain (cortex), cerebellum (Purkinje cells, black arrows) and spinal cord (motor neurons, black arrows; interneurons, white arrows) (12-week-old mice). Western blotting, immunofluorescence and immunohistochemistry have been performed using a pTUNAR custom-made antibody.

In order to identify potential microproteins involved in cellular differentiation, we looked for evolutionary conserved sORFs in lncRNAs reported to play pro-differentiation functions. For that purpose, we used PhyloCSF, a comparative genomics algorithm that determines the coding potential of a determined genomic region based on codon conservation across species (Lin et al., 2011). Our analysis revealed that TUNAR, a lncRNA with a role in pluripotency maintenance and neural differentiation, contains a 48-amino acid sORF conserved across all vertebrates (Figure 1A), that we have named pTUNAR.

TUNAR has been shown to be expressed mainly in the nervous system (Lin et al., 2014; Dong et al., 2015). Indeed, GTEx (human) and our own data (in mouse) indicate that the highest expression of the lncRNA is detected in the central nervous system (Figure 1B). In order to confirm pTUNAR translation, we analyzed already published ribosome profiling data (Wang et al., 2020) with RiboORF. This revealed that TUNAR is indeed translated in the human and mouse brain (Figure 1A). Moreover, we calculated the translational efficiency (TE) in human (TE = 1.13) and in mouse (TE = 3.72) brain and showed that it is above the median TE of regular coding genes (TE = 0.755) (Supplementary Figure S1A). *In silico* protein analysis tools predicts that pTUNAR presents a transmembrane domain in the C terminal region and that it contains several potential post-translational modifications (Supplementary Figure S1B). In fact, when overexpressing hemagglutinin (HA)-tagged pTUNAR in NIH3T3 cells, we detected a band corresponding to the predicted molecular weight of the tagged pTUNAR (7.3 KDa) and several bands with higher molecular weights, which probably correspond to the microprotein post-translationally modified (Supplementary Figure S1C). This result was confirmed by immunoprecipitation followed by western blotting (Supplementary Figure S1D).

To further confirm pTUNAR expression, we generated a polyclonal antibody, designed to recognize an epitope present in the N-terminal part of mouse pTUNAR. Using this antibody, we were able to detect specific expression of the microprotein in brain, cerebellum and spinal cord by western blotting (Figure 1C). Moreover, we detected the endogenous protein in tissue sections of these three organs by immunofluorescence (Figures 1D,F,G) and immunohistochemistry (Figure 1H). Specifically, we have detected pTUNAR in Purkinje cells (Calbindin+) in the cerebellum, in motor neurons and interneurons (NeuN+) in the spinal cord and in neurons (NeuN+) of different brain areas: cortex, hippocampus and thalamus (Figures 1D,F,G). Furthermore, we could also detect pTUNAR expression in ßIII-tubulin-positive neurons in the developing cortex, hippocampus and thalamus at embryonic day E15.5 (Figure 1E). Our results have been further supported by a recent publication showing that pTUNAR, under the name of BNLN, is also detected in pancreatic ß cells (Li et al., 2021). Altogether, we have demonstrated that TUNAR was missannotated as a lncRNA and in fact encodes for pTUNAR protein.

### pTUNAR Deficiency Improves Neural Differentiation in Mouse Embryonic Stem Cells

**FIGURE 2:**
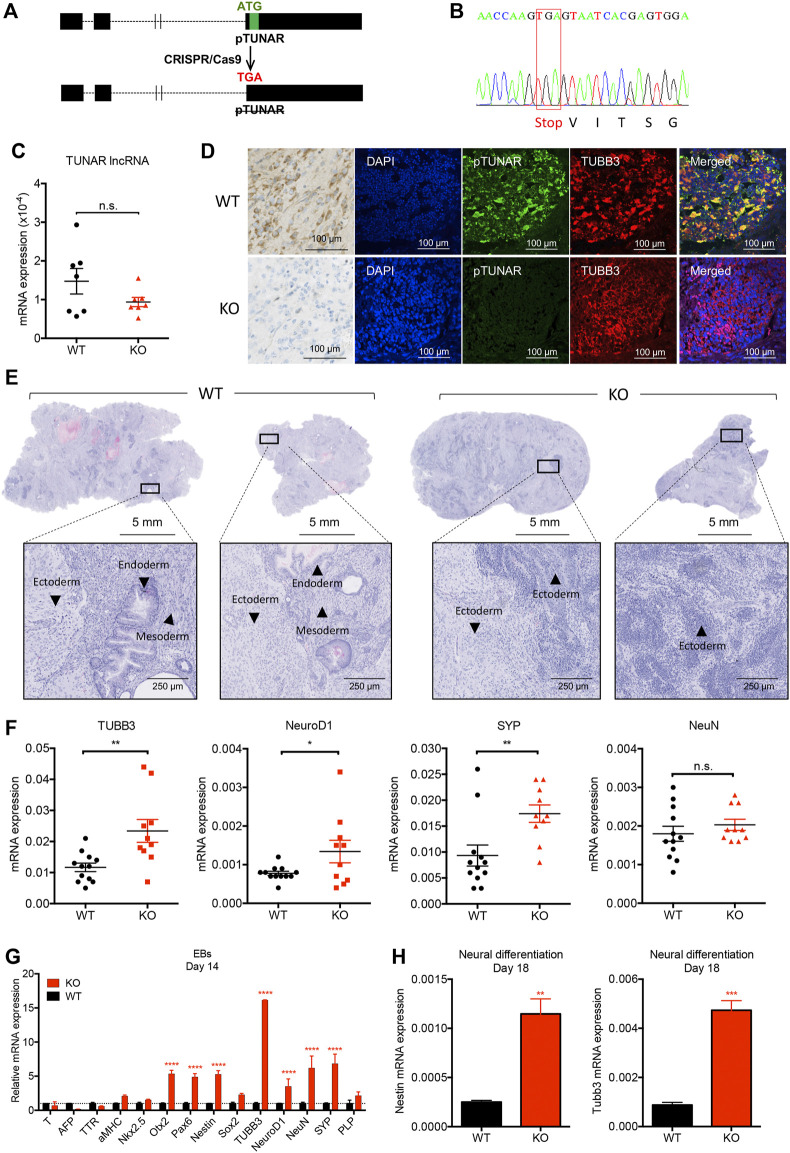
Analysis of pTUNAR deficiency in mouse embryonic stem cells (mESCs) differentiation. **(A)** Schematic representation of the pTUNAR-KO strategy. **(B)** Sequencing chromatogram showing homozygous substitution of pTUNAR ATG by a stop codon in pTUNAR-KO mESCs generated with the CRISPR/Cas9 system. **(C)** Expression analysis of TUNAR lncRNA in WT or pTUNAR-KO mESCs by qRT-PCR, normalized to GAPDH. **(D)** Immunofluorescence and immunohistochemistry images of teratomas generated with WT or pTUNAR-KO mESCs using a pTUNAR custom-made antibody. **(E)** Representative images of teratomas‘ hematoxilin and eosin staining. WT, wild type; KO, pTUNAR knock-out. **(F)** Expression analysis of the indicated genes in WT (N = 12) and pTUNAR-KO (N = 10) teratomas by qRT-PCR. Data are normalized to GAPDH. Statistical analysis is a *t*-test. * ≤ 0.05; ** ≤ 0.01; *** ≤ 0.001. **(G)** Gene expression analysis of the indicated genes by qRT-PCR in WT and pTUNAR-KO mESCs differentiated to embryoid bodies. Data are normalized to GAPDH and to control cells at day 14. Statistical analysis is a two-way ANOVA with multiple comparison. **** ≤ 0.0001. **(H)** Gene expression analysis of WT and pTUNAR-KO mESCs differentiated to neurons. Data are normalized to GAPDH. Statistical analysis is a *t*-test ** ≤ 0.01; *** ≤ 0.001.

In order to determine the function of pTUNAR in differentiation, we knocked it out in mouse embryonic stem cells (mESCs) by substituting the microprotein start codon by a stop codon using the CRISPR/Cas9 system (Figure 2A). This way, we abolished pTUNAR translation while minimally modifying the sequence of the lncRNA (Figures 2A,B). In fact, we observed that the expression of TUNAR lncRNA was not significantly affected in pTUNAR-knock out (KO) mESCs (Figure 2C). To assess differentiation potential *in vivo*, we performed a teratoma formation assay by injecting wild type (WT) mESCs or pTUNAR-KO mESCs in the flanks of immunocompromised mice. First, we could observe that WT teratomas expressed pTUNAR in neuroectoderm differentiation areas whereas pTUNAR-KO teratomas did not, validating both, the specificity of our antibody and the CRISPR-mediated KO (Figure 2D and Supplementary Figure S2A). Second, we observed that pTUNAR-KO teratomas were highly enriched in neuroectodermal differentiation areas compared to WT teratomas (Figure 2E). Indeed, by RT-qPCR we could demonstrate that they expressed significantly higher levels of neural lineage markers (Figure 2F), while we did not observe significant differences in the expression of other differentiation markers (Supplementary Figure S2B–E).

Given these results, we also tested the effect of pTUNAR overexpression in teratoma formation. Surprisingly, teratomas derived from pTUNAR overexpressing (OE) ESCs grew significantly faster, were significantly bigger at the end point (28 days) and showed significantly higher levels of Ki67 expressing cells (Supplementary Figure S2F). These evidences suggest that mESCs overexpressing pTUNAR display an increased proliferation rate *in vivo*. However, pTUNAR overexpression does not influence the differentiation to any lineage, as seen by histopathological (Supplementary Figure S2G) and gene expression analyses of teratomas (Supplementary Figure S2B–E).

To further confirm the effect of pTUNAR deficiency, we used two *in vitro* differentiation approaches. On one hand, we derived embryoid bodies (EBs) from WT and pTUNAR-KO mESCs and analyzed the expression of typical markers of the three embryonic germ layers at differentiation day 14. Interestingly, pTUNAR-KO EBs showed a significant increased expression of ectodermal lineage markers, including neural differentiation markers, compared to WT EBs (Figure 2G). On the other hand, we performed an *in vitro* mESCs neural-differentiation protocol (Mao and Zhao, 2020) and observed that pTUNAR deficiency leads to higher expression of the neuroectodermal markers *Nestin* and *ßIII-Tubulin* at differentiation day 18 (Figure 2H). Collectively, our results *in vitro* and *in vivo* indicate that pTUNAR’s deficiency in mESCs promotes differentiation towards the neural lineage.

### pTUNAR Overexpression Impairs Neurite Outgrowth

**FIGURE 3:**
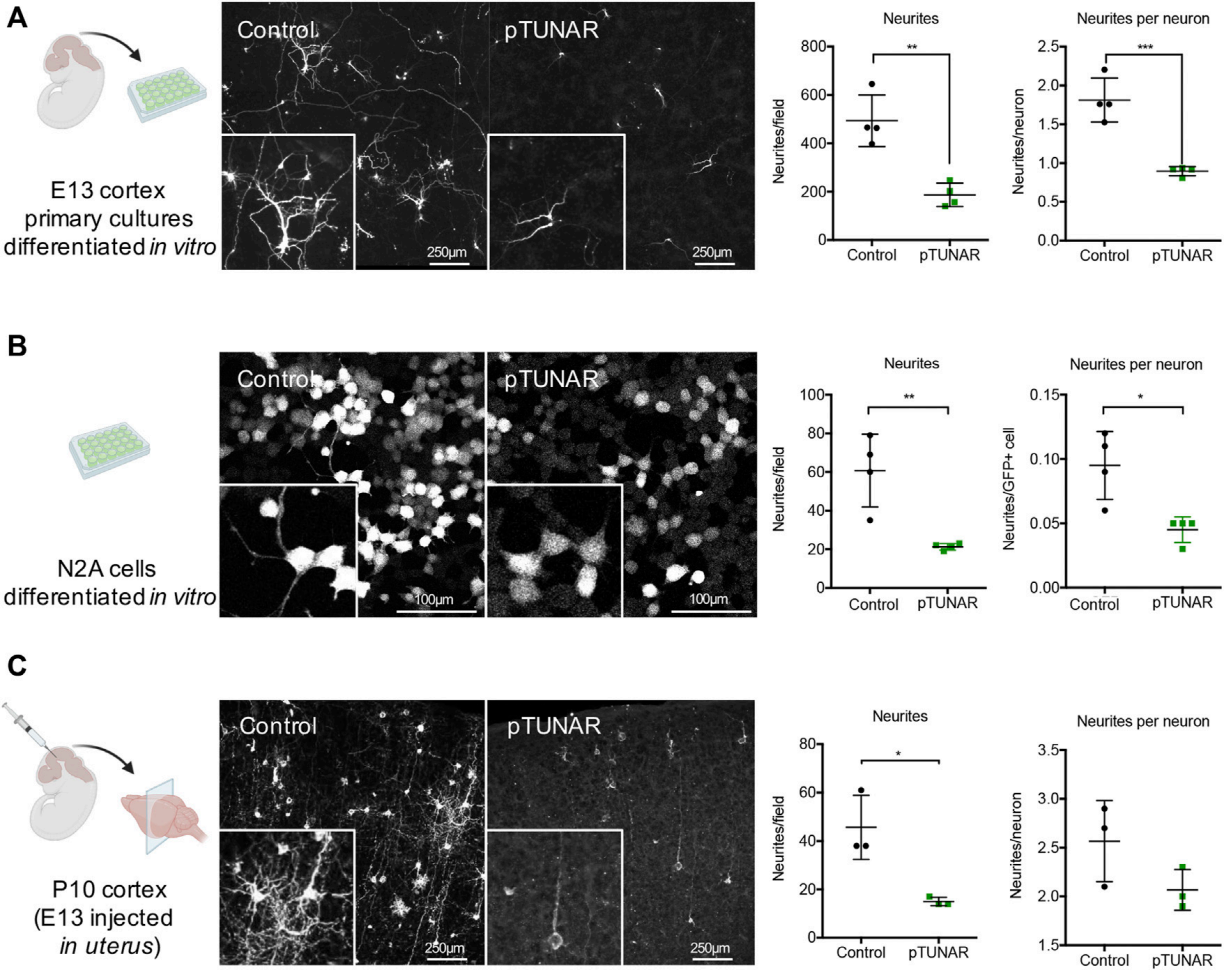
Study of pTUNAR’s role in neurite formation. **(A)** Schematic representation of the experiment: E13 cortex primary cultures, transfected with a CAG-GFP or a CAG-pTUNAR-IRES-GFP plasmid and differentiated for 7 days *in vitro* **(left).** Representative images of the cells stained with a GFP antibody **(middle).** Quantification of the neurites observed in different fields and proportion of neurites/neuron **(right).** **(B)** Schematic representation of the experiment: N2A cells transduced with lentiviral vectors encoding either GFP or pTUNAR-IRES-GFP and differentiated for 72 h with serum deprivation (1% FBS) **(left).** Representative images of cells stained with a GFP antibody **(middle).** Quantification of the neurites observed in different fields and proportion of neurites/neuron **(right).** **(C)** Schematic representation of the experiment: P10 cortex developed after *in uterus* injection at embryonic day E13 with a retroviral vector encoding RFP and a lentiviral vector encoding pTUNAR-IRES-GFP **(left).** Representative images of cortical neurons stained with RFP (control) and a GFP (pTUNAR) antibodies **(middle).** Quantification of the neurites observed in different fields and proportion of neurites/neuron **(right).** Statistical analysis is a *t*-test * ≤ 0.05; ** ≤ 0.01; *** ≤ 0.001. The illustrations in this figure were created using BioRender.com.

To pinpoint pTUNAR’s role in neural differentiation, we perform gain-of-function studies in *vitro* and *in vivo* mouse neural development systems. First, we used an *in vitro* system of primary cells dissociated from E13 cerebral cortex, transfected with a pTUNAR-overexpressing plasmid (or control plasmid) and differentiated for 7 days (Supplementary Figure S3A). We analysed the proliferation of these cells 36 h after transfection by Ki67 immunostaining or BrdU incorporation and found no differences between control and pTUNAR-overexpression (Supplementary Figure S3B). Then, we analyzed the expression of different brain cell type markers (NeuN for neurons, Olig2 for oligodendrocytes, GFAP for astrocytes and Nestin for neural stem cells) and did not observe any significant difference after 7 days of differentiation either (Supplementary Figure S3C). However, pTUNAR-overexpressing neurons appeared less mature. Indeed, quantifying their neurites revealed a significantly lower number of neurites in total and significantly lower numbers of neurites per neuron (Figure 3A). The same results were observed after transducing, instead of transfecting, these cells with lentiviral vectors (Supplementary Figure S3D). Moreover, a Scholl analysis indicated that there is a defect in arborization in pTUNAR overexpressing cells (Supplementary Figure S3E). We also examined this phenotype in Neuro-2a (N2A) cells, a mouse neuroblastoma cell line commonly used for neurite formation assays, and confirmed the same phenotype: pTUNAR overexpressing cells had both, significantly lower number of neurites and a significantly lower number of neurites per cell (Figure 3B). To further characterize this phenotype *in vivo*, we performed *in utero* intracranial injections of a retroviral vector expressing RPF (control vector) and a lentiviral vector expressing pTUNAR-IRES-GFP in E13 embryos and analyzed their brains postnatally at day 10. Again, we observed that cortical neurons overexpressing pTUNAR had significantly fewer neurites and also fewer neurites per neuron (Figure 3C and Supplementary Figure S3F). Of note, neurite length was similar between control and pTUNAR overexpression condition in all *in vivo* and *in vitro* experiments (Supplementary Figure S3G), suggesting that there is a defect in neurite branching rather than in extension. Altogether, these results indicate that pTUNAR inhibits neurite formation *in vitro* and *in vivo*.

### pTUNAR Microprotein Interacts With SERCA2 and Regulates Intracellular Calcium Exchange

**FIGURE 4:**
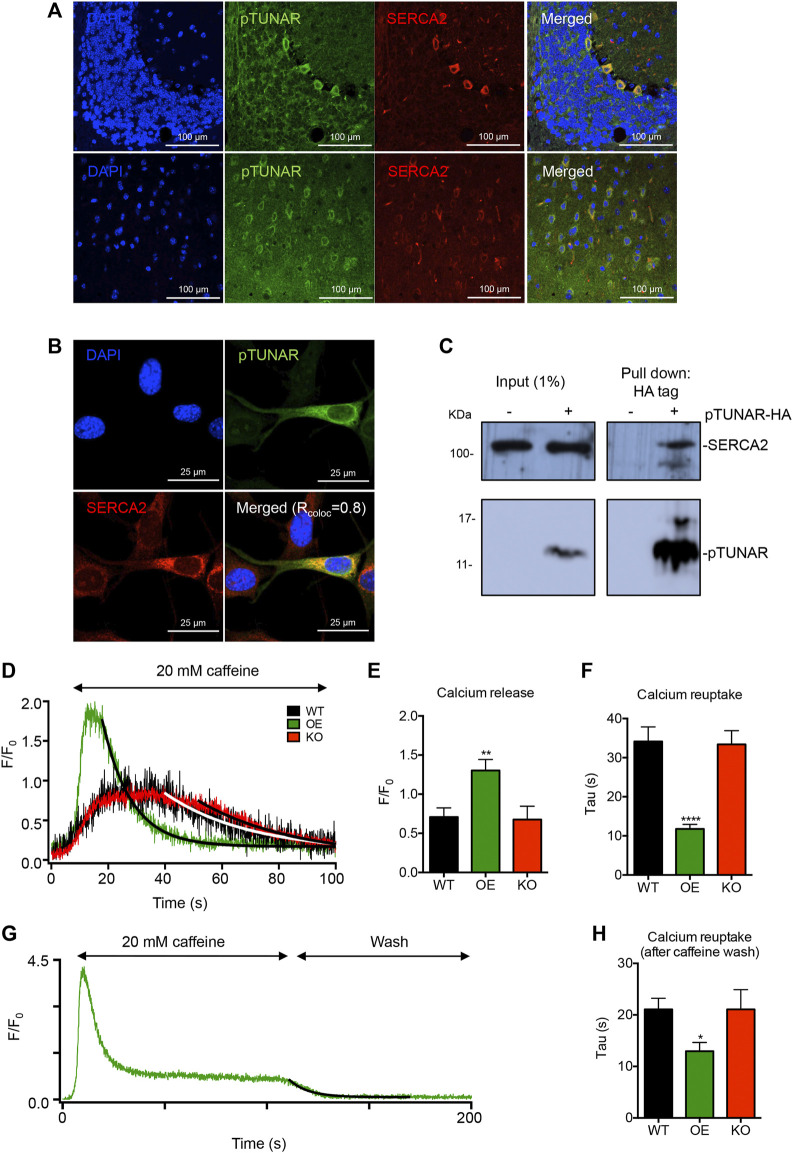
pTUNAR subcellular localization and molecular characterization. **(A)** Immunofluorescence images showing expression of pTUNAR and SERCA2 in adult cerebellum **(upper panel)** and cerebral cortex **(lower panel)** (12 weeks old). Immunofluorescence has been performed using a pTUNAR custom-made antibody. **(B)** Immunofluorescence images of NIH3T3 cells transduced with an inducible lentiviral vector expressing HA-tagged pTUNAR. Expression was induced with 1 μg/ml of doxycycline for 72 h. Cells are co-stained with an HA tag antibody and a SERCA2 antibody. Co-localization analysis was perfomed with Fiji (ImageJ) and the co-localization coefficient (R_coloc_) shown is the average of 6 different cells. **(C)** Immunoprecipitation of HA-tagged pTUNAR followed by western blotting in NIH3T3 cells transduced with an inducible lentiviral vector expressing HA tagged pTUNAR or a control vector. Expression was induced with 1 μg/ml of doxycycline for 72 h. Membranes were incubated with an HA tag antibody and a SERCA2 antibody. **(D)** Representative calcium transients elicited by caffeine in differentiated wild-type mESCs (WT, black), mESCs overexpressing pTUNAR (OE, green) or mESCs pTUNAR-KO (KO, red). Solid lines represent fits with an exponentially decaying function. **(E)** Calcium release measured by the amplitude of the caffeine-induced calcium transient in differentiated WT, pTUNAR OE and pTUNAR-KO mESCs. **(F)** Calcium reuptake measured by analyzing the Tau for the decay of the caffeine induced calcium transient in differentiated WT pTUNAR OE and pTUNAR-KO mESCs. **(G)** Representative image of the kinetics of the calcium trace during exposure to caffeine and when caffeine is washed off in differentiated pTUNAR OE mESCs. To estimate calcium removal from the cytosol during the wash phase, this part of the traces was fitted with an exponentially decaying function. **(H)** Impact of pTUNAR overexpression or deficiency on tau during the wash phase. Statistical analysis is a one-way ANOVA with a Dunnet correction for multiple comparisons. * ≤ 0.05; ** ≤ 0.01; **** ≤ 0.0001.

In order to unravel the mechanism behind pTUNAR’s function, we first determined its subcellular localization. Given the perinuclear localization of pTUNAR observed by previous experiments (Figures 1D–H), we tested whether pTUNAR was located in the endoplasmic reticulum (ER). Indeed, we could observe that pTUNAR co-localize with the Sarco/Endoplasmic Reticulum Calcium ATPase (SERCA2) in cerebellum and brain tissue by immunostaining (Figure 4A). To further confirm this result, we overexpressed a C-terminal domain HA-tagged pTUNAR in NIH3T3 mouse fibroblasts and performed immunofluorescence with typical markers of different organelles. As expected, we found a strong co-localization of pTUNAR with SERCA2 (Figure 4B). Interestingly, we could also detect pTUNAR in small and large extracellular vesicles (Supplementary Figure S4B). We did not observe co-localization of pTUNAR with DAPI (nucleus), AIF (mitochondria), 58 K (Golgi apparatus) and LAMP2 (lysosomes) (Supplementary Figure S4A). Importantly, by co-immunoprecipitation experiments we could demonstrate that pTUNAR and SERCA2 physically interact (Figure 4C). Given the role of SERCA2 as a transporter of calcium from the cytoplasm to the endoplasmic reticulum, we decided to examine the effect of pTUNAR in the regulation of intracellular calcium. We differentiated WT mESCs, pTUNAR overexpressing (OE) mESCs or pTUNAR-KO mESCs to neurons (Mao and Zhao, 2020) and tested their capacity to uptake and release calcium from the ER. For this purpose, we used caffeine to induce calcium release from the ER and measured the resulting calcium transient and its decay as calcium was eliminated from the cytosol by reuptake. We observed that there was a significant increase in calcium release (shown by a higher amplitude (F/F_0_)) in cells overexpressing pTUNAR (Figures 4D,E), likely indicating that pTUNAR OE cells have higher concentrations of calcium in the ER. We also observed that calcium reuptake was significantly faster (reflected by the lower tau for the decay of the calcium transient) in pTUNAR overexpressing cells, not only while caffeine was still present (Figures 4D,F), but also when it was removed (Figures 4G,H). Altogether, these results indicate that pTUNAR modulates intracellular calcium dynamics increasing the efficiency by which calcium is removed from the cytoplasm, probably through its interaction with SERCA2.

## Discussion

In this manuscript, we describe a novel 48-amino acid lncRNA-encoded microprotein, pTUNAR, which is expressed in the central nervous system. pTUNAR defficiency in mouse embryonic stem cells promotes their differentiation towards the neural lineage both *in vitro* and *in vivo*. Moreover, pTUNAR overexpression in different models of neural differentiation impairs the formation of neurites. pTUNAR localizes in the membrane of the endoplasmic reticulum where it co-localizes and interacts with SERCA2, an ATPase that pumps calcium from the cytoplasm to the ER. pTUNAR overexpression increases the calcium stored in the ER and accelerates calcium clearance from the cytoplasm, indicating that pTUNAR acts as a modulator of intracellular calcium dynamics. Interestingly, another group has independently found that pTUNAR, which they named BNLN, interacts with another member of the SERCA family (Li et al., 2021). In particular, they showed that pTUNAR/BNLN interacts with the islet ß cell-specific SERCA3 and, in response to high glucose, decreases the Ca^2+^ reuptake capacity from the ER. This is consistent with pTUNAR acting as a modulator of calcium dynamics via SERCA family proteins, which seems to be context and cell-type dependent. Further research is needed to understand the role of pTUNAR regulating calcium dynamics and its relationship with the SERCA family.

Our ribosome sequencing analysis indicates that, in addition to pTUNAR, there could be a longer microprotein encoded by TUNAR lncRNA which starts in an ATG upstream pTUNAR’s start codon. This longer isoform has 65 amino acids and shares the C terminal domain- including the transmembrane region- with pTUNAR. The C terminal region, shared by both isoforms, is conserved among all vertebrates, from humans to zebrafish. However, the initial 17 amino acids of the long isoform are less conserved throughout evolution. Moreover, comparing the ribosome profiling reads of the first 17 amino acids of the long isoform with the first 17 amino acids of the short isoform (pTUNAR), there are four times more reads in the latter (data not shown). As the long isoform contains the short isoform, we could say that the 48-amino acid microprotein is three times more translated than the 65-amino acid protein. This reasoning, together with the phylogenetic conservation, made us focus on pTUNAR rather than on longer isoform. However, it is important and interesting to consider that TUNAR lncRNA could code for a 65-amino acid isoform which could be translated in a time or tissue-specific manner.

The Sarco/Endoplamic Reticulum Calcium ATPase (SERCA2) is regulated by several microproteins. On the one hand, there is a family of SERCA-inhibiting microproteins, called regulins, which are master regulators of calcium signaling in muscle (Anderson et al., 2016). It is well known that Phospholamban (PLN) and Sarcolipin (SLN) inhibit the activity of SERCA2a in the heart (MacLennan et al., 2003). Myoregulin (MLN), a lncRNA-encoded microprotein (Anderson et al., 2015), has been shown to inhibit SERCA in skeletal muscle. Moreover, two other regulins, endoregulin (ELN) and another-regulin (ALN), have been shown to inhibit SERCA in non-muscle cells (Anderson et al., 2016). On the other hand, DWORF is a microprotein that has been shown to act as a SERCA activator in muscle by displacing the regulins PLN, SLN and MLN (Nelson et al., 2016). However, it has not been described to date any microprotein regulating calcium dynamics in the nervous system. We have shown that pTUNAR, which is expressed in the central nervous system, interacts with SERCA2 and improves calcium reuptake from the cytoplasm. pTUNAR’s transmembrane domain does not contain the xLFxxF motif shared by all regulins (Anderson et al., 2016) and does not display apparent amino acid similarity with regulins or with DWORF (Supplementary Figure S4C,D). However, from a functional point of view, our results suggest that pTUNAR is an activator of SERCA2, such as DWORF. We cannot dismiss the possibility that pTUNAR also improves the calcium clearance from the cytoplasm by other mechanisms (e.g., by regulating the plasma membrane Ca^2+^ ATPase).

Calcium signaling has a very important role in the nervous system since it regulates neuronal differentiation, neurotransmitter phenotype specification, dendritic arborization and axon outgrowth and path finding (Rosenberg and Spitzer, 2011). In amphibians, spontaneous elevations of intracellular calcium occur during gastrulation in dorsal ectodermal cells, which are the cells where neural specification take place (Leclerc et al., 2000). In mouse embryonic stem cells, an increase of intracellular calcium is related with the generation of neuroectodermal cells and neurons (Lin et al., 2010). Together, these data indicate that elevated levels of calcium in the cytoplasm induce neural differentiation (Leclerc et al., 2012). Our results show that pTUNAR deficiency promotes neural differentiation from mESCs. It is conceivable that in the absence of pTUNAR, the activity of SERCA2 is reduced, thereby impairing calcium storage in the ER and increasing cytosolic calcium that may favor neural differentiation.

On the other hand, neurite formation depends on a fine-tuned regulation of intracellular calcium. Hui and others demonstrated that if calcium concentration in the cytoplasm is too low, neurites do not grow and, if is too high, neurite’s growth stalls (Hui et al., 2007). We observe that pTUNAR overexpression impairs neurite formation in different models of neuritogenesis. This could be explained by an increase in calcium reuptake triggered by pTUNAR (Figures 4C–G), which induces a decrease in cytoplasmic calcium levels, impairing in turn neurite formation.

As a final remark, it has been shown that TUNAR lncRNA is downregulated in glioblastoma multiforme patient’s samples (Reon et al., 2016). Loss of calcium homeostasis has been suggested as one of the mechanisms that contribute to tumor growth (Leclerc et al., 2016) as well as therapy resistance and metastasis (Maklad et al., 2019) in brain cancers. Thus, it is possible that the downregulation of pTUNAR microprotein might contribute the dysregulation of calcium dynamics in these deadly tumors.

In summary, we have discovered and characterized a new lncRNA-encoded microprotein with a role in neural differentiation through the regulation of intracellular calcium. From a more general point of view, we have uncovered that the microproteome regulates neural differentiation, and could hide important players involved in neural homeostasis and disease that remain to be identified.

## Data Availability

The original contributions presented in the study are included in the article/Supplementary Material, further inquiries can be directed to the corresponding authors.
