# Supplementary material for: TUNAR lncRNA Encodes a Microprotein that Regulates Neural Differentiation and Neurite Formation by Modulating Calcium Dynamics
Source: Front Cell Dev Biol. 2021 Dec 31;9:747667. doi: 10.3389/fcell.2021.747667 (PMC8758570; doi:10.3389/fcell.2021.747667)
Supplement: Supplementary file 1 [file Presentation1.pdf]

Supplementary Material

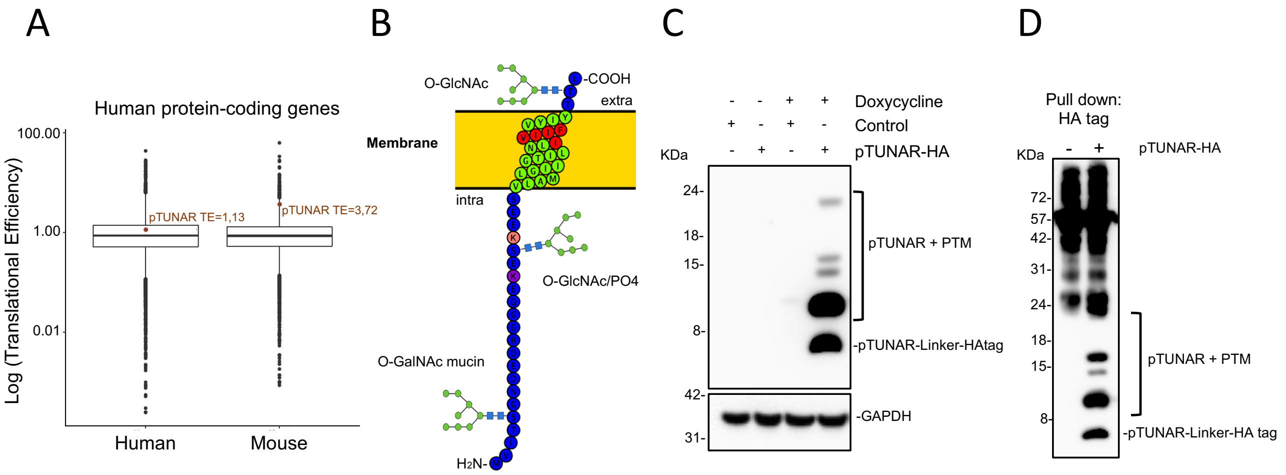

Supplementary Figure 1. pTUNAR translation and its molecular features (A) Boxplot showing the translational efficiency (TE) of human and mouse pTUNAR compared to the mean TE of regular protein-coding genes. **(B)** Schematic representation of pTUNAR (predicted with Protter software). Amino acids marked in red correspond to a predicted SUMO interaction motif (analyzed by GPS-Sumo 2.0). Lysine marked in purple is predicted to be ubiquitinated (analyzed by UbPred). Lysine marked in orange is predicted to be ubiquitinated (analyzed by UbPred) and/or sumoylated (analyzed by GPS-Sumo 2.0). Serines and threonines with schematic representations of sugars attached are predicted to be glycosylated with the indicated moieties (analyzed by glycomics tools from Expasy). **(C)** Western blotting of NIH3T3 transduced with an inducible lentiviral vector encoding HA-tagged pTUNAR or a control vector. Expression was induced with 1µg/ml of doxycycline for 72 hours. Membranes were incubated with an HA tag antibody and a GAPDH antibody as a loading control. **(D)** Immunoprecipitation of pTUNAR using and anti-HA antibody in NIH3T3 transduced with an inducible lentiviral vector encoding HA-tagged pTUNAR or a control vector and analyzed by western blotting. Membranes were incubated with an HA tag antibody. PTM, post-translational modifications.

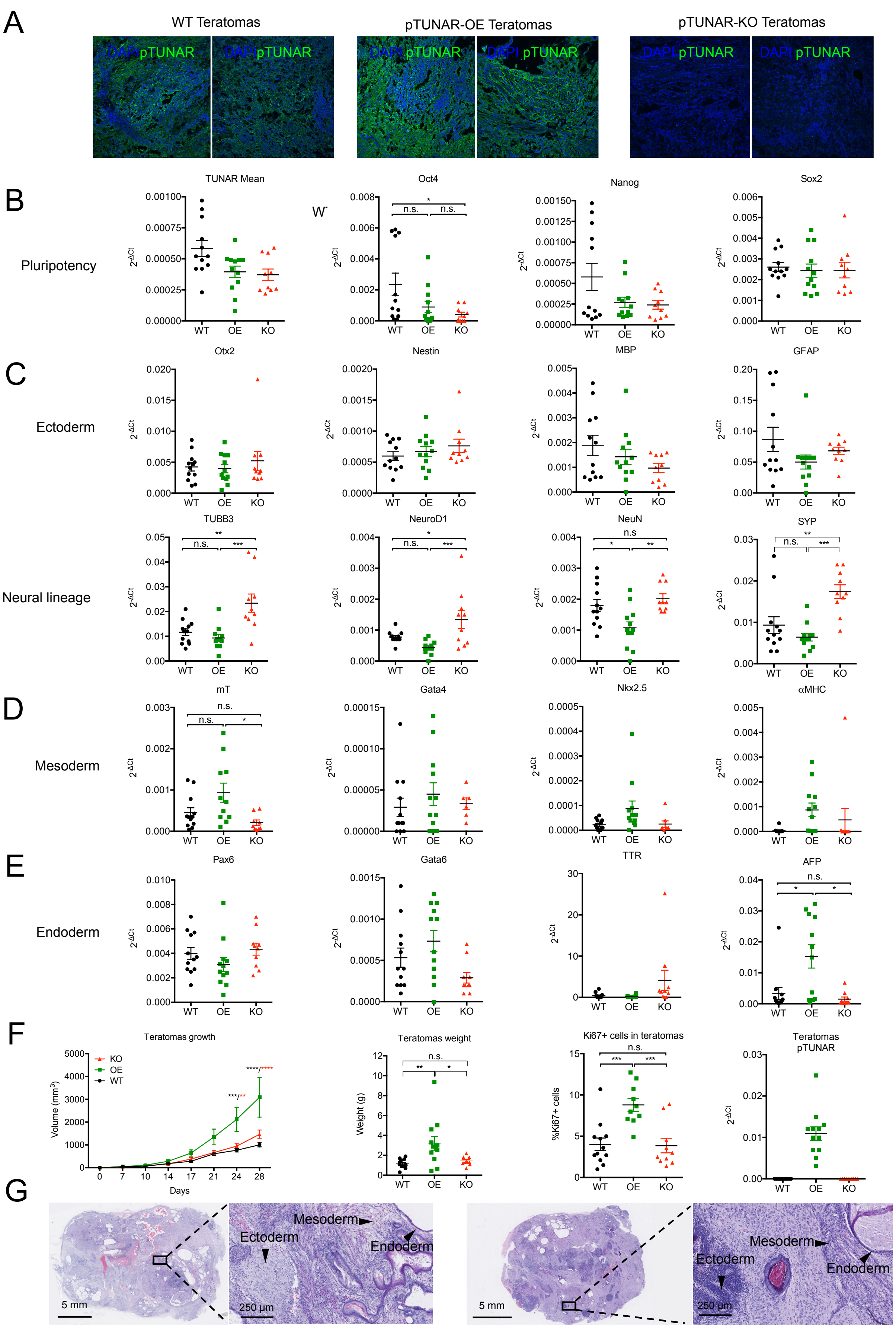
A
WT Teratomas
pTUNAR-OE Teratomas
pTUNAR-KO Teratomas
DAPI pTUNAR
B
Pluripotency
TUNAR Mean
Oct4
Nanog
Sox2
C
Ectoderm
Otx2
Nestin
MBP
GFAP
Neural lineage
TUBB3
NeuroD1
NeuN
SYP
D
Mesoderm
mT
Gata4
Nkx2.5
αMHC
E
Endoderm
Pax6
Gata6
TTR
AFP
F
Teratomas growth
KO
OE
WT
Volume (mm³)
Days
Teratomas weight
Weight (g)
Ki67+ cells in teratomas
%Ki67+ cells
Teratomas pTUNAR
$2^{-\Delta Ct}$
WT
OE
KO
n.s.
G
Ectoderm
Mesoderm
Endoderm
5 mm
250 μm

Supplementary Figure 2. Analysis of pTUNAR deficiency in mouse Embryonic Stem Cells (mESCs) differentiation. (A) Immunofluorescence images of teratomas generated with WT, pTUNAR-OE or pTUNAR-KO mESCs using a pTUNAR antibody. Images taken with a C2 confocal microscope (Nikon) at 20x magnification. **(B-E)** Expression analysis of the indicated genes in teratomas by qRT-PCR. Data are normalized to GAPDH. Statistical analysis is a one-way ANOVA with a Dunnet correction for multiple comparisons. *≤0.05. **(F)** From left to right: analysis of teratomas' growth over time; teratomas' weight at day 28, percentage of Ki67+ cells in teratomas at day 28; pTUNAR expression in teratomas at day 28. pTUNAR expression was measured by qRT-PCR and normalized to GAPDH. **(G)** Representative images of hematoxilin and eosin stainings of teratomas generated with mESCs overexpressing pTUNAR.

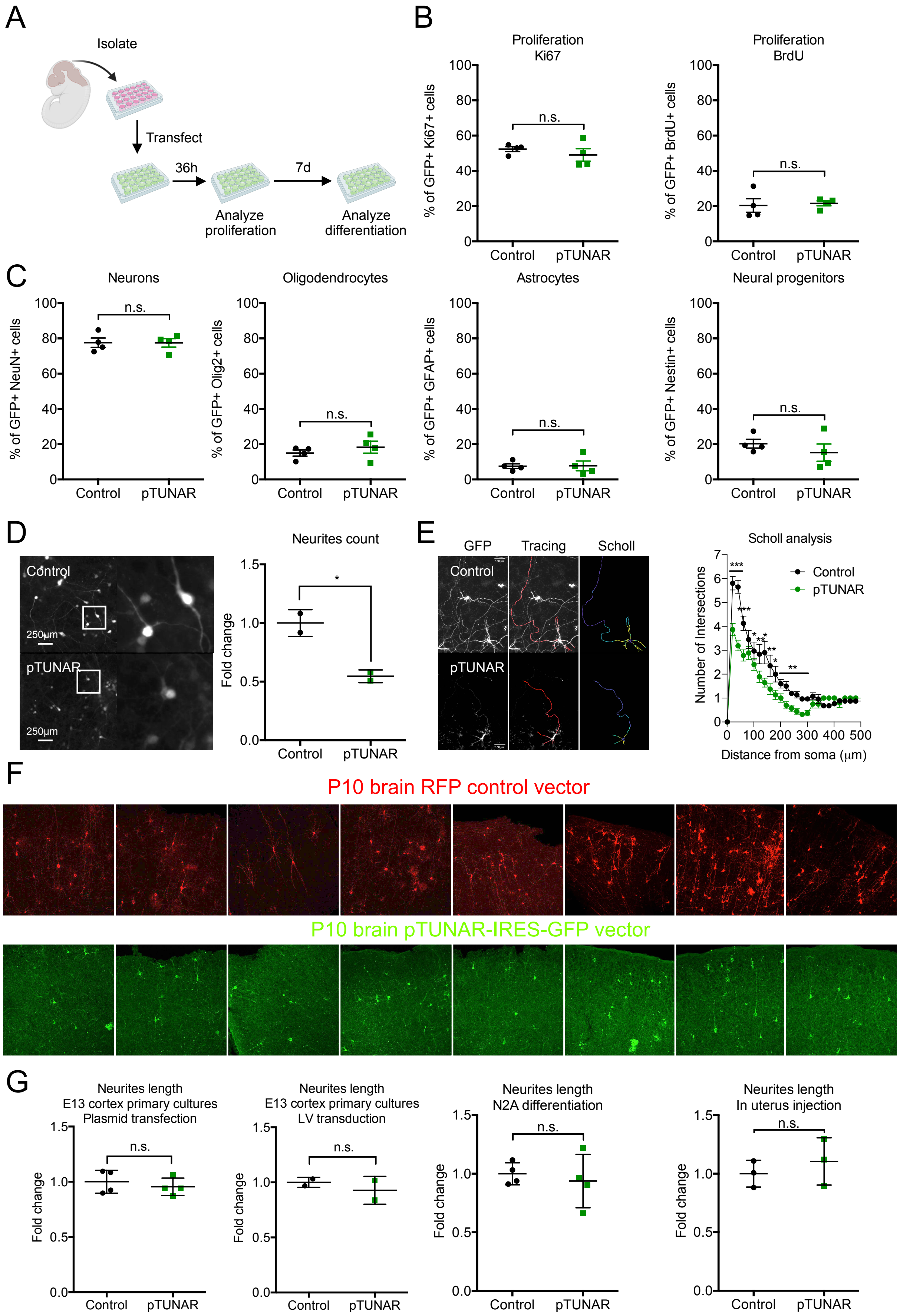
A
Isolate
Transfect
36h
7d
Analyze proliferation
Analyze differentiation
B
Proliferation Ki67
% of GFP+ Ki67+ cells
Proliferation BrdU
% of GFP+ BrdU+ cells
n.s.
Control
pTUNAR
C
Neurons
% of GFP+ NeuN+ cells
Oligodendrocytes
% of GFP+ Olig2+ cells
Astrocytes
% of GFP+ GFAP+ cells
Neural progenitors
% of GFP+ Nestin+ cells
D
Control
pTUNAR
250µm
Neurites count
Fold change
*
E
GFP
Tracing
Scholl
Scholl analysis
Number of Intersections
Distance from soma (µm)
F
P10 brain RFP control vector
P10 brain pTUNAR-IRES-GFP vector
G
Neurites length
E13 cortex primary cultures
Plasmid transfection
LV transduction
N2A differentiation
In uterus injection
Fold change

Supplementary Figure 3. pTUNAR's role in neurite formation. (A) Schematic representation of the experiment: E13 cortex primary cultures were transfected with a CAG-GFP or a CAG-pTUNAR-IRES-GFP plasmid. Proliferation was analyzed 36 hours after transfection and differentiation was analyzed 7 days after transfection. The illustration was created with Biorender.com **(B)** Analysis of the proliferation of E13 cortex primary cultures transfected with a CAG-GFP or a CAG-pTUNAR-IRES-GFP plasmid. Cells were stained with a GFP antibody and with a Ki67 (left) or a BrdU antibody (right) 36 hours after transfection and quantified with Fiji (Image J). Individual values represent independent fields. **(C)** Analysis of neural lineage markers in E13 cortex primary cultures, transfected with a CAG-GFP or a CAG-pTUNAR-IRES-GFP plasmid and differentiated for 7 days *in vitro*. Cells were stained with a GFP antibody and with a NeuN (neurons), Olig2 (oligodendrocytes), GFAP (astrocytes) or Nestin (neural progenitors) antibodies and quantified with Fiji (Image J). Individual values represent independent fields. **(D)** E13 cortex primary cultures were transduced with a CAG-GFP or a CAG-pTUNAR-IRES-GFP lentiviral vector and differentiated for 7 days *in vitro*. Left, representative images of the cells stained with a GFP antibody. Right, quantification of the number of neurites observed in different fields represented as fold change compared to the control. **(E)** Scholl analysis of E13 cortex primary cultures, transfected with a CAG-GFP or a CAG-pTUNAR-IRES-GFP plasmid and differentiated for 7 days *in vitro*. 37 cells from each condition were analyzed using the Scholl analysis tool from ImageJ. Left, representative images; right, graph with the Scholl analysis results **(F)** Images of P10 cortical neurons developed after *in uterus* injection at embryonic day E13 with a retroviral vector encoding RFP and a lentiviral vector encoding pTUNAR-IRES-GFP, and stained with an RFP (control) and a GFP (pTUNAR) antibodies. **(G)** Measurements of neurite length in the indicated neurite formation experiments (represented as fold change compared to the control).

A

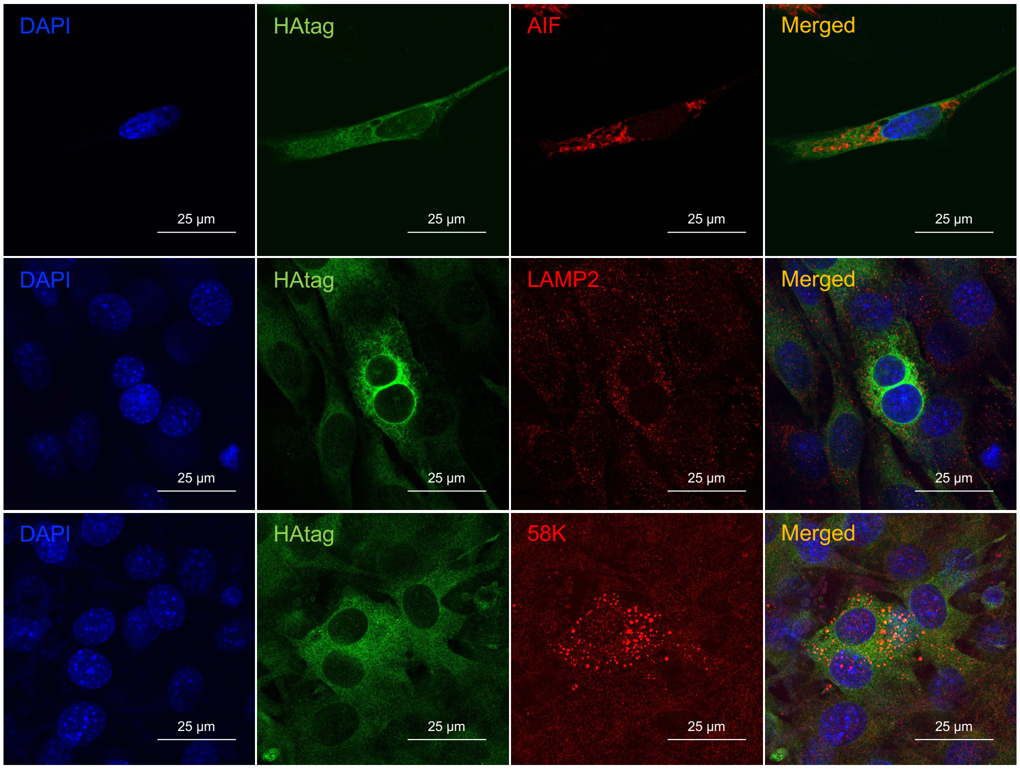

B

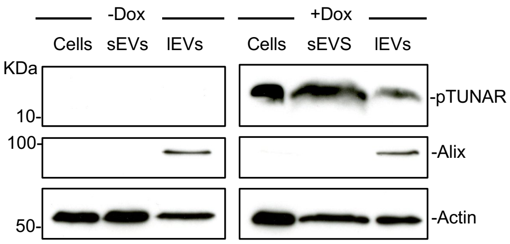

C

```
                                                                 Transmembrane domain
> ALN     MEVSQAASGTDGVRERRGSFEAGRRNQDEAPQSGMNGLPKHSYWLDLWLFILFDLALFVFVYLLP-65
> ELN                 MGQMVPPRSIQNEDFWKNPWDVGGLTVIGLFTSTFLLFVLFAVVFGYVEKAVFEEE-56
> PLN                       MEKVQYLTRSAIRRASTIEMPQQARQNLQNLFINFCLILICLLLICIIVMLL-52
> MLN                         MSGKSWVLISTTSPQSLEDEILGRLLKILFVLFVDLMSIMYVVITS-46
> SLN                                             MERSTQELFINFTVVLITVLLMWLLVRSYQY-31
> SCL                                              MSEARNLFTTFGILAILLFFLYLIYAVL-28
> pTUNAR                           MVITSGNDEDRGGQEKESKEESGIIGTILNLIVIIFVYIYTTL-48
```

D

```
                                                                 Transmembrane domain
> DWORF                                        MAEKESTSPHLMVPILLLVGWIVGCIIVIYIVFF-34
> pTUNAR                           MVITSGNDEDRGGQEKESKEESGIIGTILNLIVIIFVYIYTTL-48
```

Supplementary Figure 4. pTUNAR's subcellular localization and comparison with other SERCA-regulator proteins. (A) Immunofluorescence images of NIH3T3 cells transduced with an inducible lentiviral vector expressing HA-tagged pTUNAR. Cells are co-stained with an HA tag antibody and with an AIF (mitochondria), LAMP2 (lysosomes) or 58K (Golgi apparatus) antibody. **(B)** Western blotting of extracellular vesicles secreted by NIH3T3 cells transduced with an inducible lentiviral vector expressing HA-tagged pTUNAR or a control vector. Membranes were incubated with an HA tag antibody and an Alix antibody as exosomes loading control, and an Actin antibody as a general loading control. sEVs, small extracellular vesicles; lEVs, large extracellular vesicles. **(C)** Alignment of the amino acid sequence of pTUNAR with the proteins of the regulin family. Green columns indicate the amino acids that are identically conserved in all regulins; blue columns indicate the amino acids that are weakly similar in all regulins (based on Anderson, 2016). **(D)** Alignment of the amino acid sequence of pTUNAR and DWORF microprotein.

**Primer Name**	**Sequence**
HAtag_qPCR_R	TCCGGCACATCATACGGATA
hmOtx2_F	GAATCCAGGGTGCAGGTATGG
hmOtx2_R	CTGAACTCACTTCCCGAGCTG
mAFP_F	TCGTATTCCAACAGGAGG
mAFP_R	AGGCTTTTGCTTCACCAG
mGAPDH_2_F	TGTGTCCGTCGTGGATCTGA
mGAPDH_2_R	TTGCTGTTGAAGTCGCAGGAG
mGATA4_F	GAAAACGGAAGCCCAAGAACC
mGATA4_R	TGCTGTGCCCATAGTGAGATGAC
mGata6_F	TCATTACCTGTGCAATGCATGCGG
mGata6_R	ACGCCATAAGGTAGTGGTTGTGGT
mGFAP_F	CGTTAAGCTAGCCCTGGACA
mGFAP_R	GGATCTGGAGGTTGGAGAAAG
mMBP_F	CTATAAATCGGCTCACAAGG
mMBP_R	AGGCGGTTATATTAAGAAGC
mNANOG_F	CAAGGGTCTGCTACTGAGATGCTCTG
mNANOG_R	TTTTGTTTGGGACTGGTAGAAGAATCAG
mNestin_F	TCAGATCGCTCAGATCCTGG
mNestin_R	TTCTCAGCCTCCAGCAGAGT

mNeuN_F	ATCGTAGAGGGACGGAAAATTGA
mNeuN_R	GTTCCCAGGCTTCTTATTGGTC
mNeuroD1_F	ATGACCAAATCATACAGCGAGAG
mNeuroD1_R	TCTGCCTCGTGTTCCTCGT
mNkx2.5_F	AGCAACTTCGTGAACTTTG
mNkx2.5_R	CCGGTCCTAGTGTGGA
mOCT4_F	GTTGGAGAAGGTGGAACCAA
mOCT4_R	CCAAGGTGATCCTCTTCTGC
mPax6_F	AGTGAATGGGCGGAGTTATG
mPax6_R	ACTTGGACGGGAACTGACAC
mPLP_F	AGCAAAGTCAGCCGCAAAAC
mPLP_R	CCAGGGAAGCAAAGGGGG
mSOX2_F	CGTAAGATGGCCCAGGAGAA
mSOX2_R	GCTTCTCGGTCTCGGACAAA
mSynaptophysin_F	CAGTTCCGGGTGGTCAAGG
mSynaptophysin_R	ACTCTCCGTCTTGTTGGCAC
mT_F	GCTTCAAGGAGCTAACTAACGAG
mT_R	CCAGCAAGAAAGAGTACATGGC
mTTR_F	CTCACCACAGATGAGAAG
mTTR_R	GGCTGAGTCTCTCAATTC

mTUBB3_new_F	TAGACCCCAGCGGCAACTAT
mTUBB3_new_R	GTTCCAGGTTCCAAGTCCACC
mTUNAR_F	GCCTCCGGATGCTCTTCTC
mTUNAR_R	CGGTCTTCATCGTTTCCACT
mTUNAR1_qPCR__F	CGATGAAGACCGGGGAGG
mα-MHC_F	ACCGTGGACTACAACAT
mα-MHC_R	CTTTCGCTCGTTGGGA

Supplementary Table 1. Primers used in this study.

Antibody Name	Species	Host	Application	Dilution	Company	Ref. Nº
58K Golgi protein (58K-9)	Mouse	Mouse	IF	1:100	Novus Biologicals	NB600-412SS
AIF	Mouse	Mouse	IF	1:100	Santa Cruz	SC-13116
b3-tubulin (TU-20)	Mouse	Mouse	IF	1:100	Santa Cruz	SC-51670
Calbindin	Mouse	Mouse	IF	1:500	Swant	CB300
FLAG tag	Mouse	Mouse	WB	1:2000	Sigma	F1804
GAPDH	Mouse	Mouse	WB	1:10000	ThermoFisher	AM4300
HA tag	Mouse	Rabbit	WB	1:5000	Abcam	Ab9110
HA tag	Mouse	Rabbit	IF/IP	1:150 (IF)	Sigma	H6908
LAMP-2/CD107b	Mouse	Mouse	IF	1:1000	Novus Biologicals	NBP2-22217SS
NeuN	Mouse	Mouse	IF	1:100	Merck	MAB377
pTUNAR	Mouse	Rabbit	WB/IF/IHC	1:500(WB)<br>1:10(IF)<br>1:500(IHC)	Proteogenix	Custom-made
SERCA2 ATPase	Mouse	Mouse	IF/WB	1:100(IF)<br>1:1000(WB)	Novus Biologicals	NB300-581-0,01ml

Supplementary Table 2. Antibodies used in this study.
